# Adrenal Insufficiency Associated With Empty Sella Syndrome and Steroid Malabsorption Complicated With Septic Shock Due to Post-transplant Pyelonephritis: A Case Report

**DOI:** 10.7759/cureus.38234

**Published:** 2023-04-28

**Authors:** Hisato Shima, Keiko Miya, Kazuyoshi Okada, Toshio Doi, Jun Minakuchi

**Affiliations:** 1 Kidney Disease, Kawashima Hospital, Tokushima, JPN; 2 Internal Medicine, Kawashima Hospital, Tokushima, JPN

**Keywords:** infection, secondary adrenal insufficiency, empty sella syndrome, hyponatremia, renal transplantation

## Abstract

Renal transplant recipients are immunocompromised and predisposed to develop hyponatremia because they are exposed to immunological, infectious, pharmacological, and oncologic disorders. A 61-year-old female renal transplant recipient was admitted with diarrhea, anorexia, and headache for about a week during the tapering of oral methylprednisolone for chronic renal allograft rejection. She also presented hyponatremia and was suspected to have secondary adrenal insufficiency based on a low plasma cortisol level of 1.9 μg/dL and a low adrenocorticotropic hormone level of 2.6 pg/mL. Brain magnetic resonance imaging to assess the hypothalamic-pituitary-adrenal axis revealed an empty sella. She also developed septic shock and disseminated intravascular coagulation due to post-transplant pyelonephritis. She had reduced urine output and underwent hemodialysis. Both plasma cortisol and adrenocorticotropic hormone levels were relatively low (5.2 μg/dL and 13.5 pg/mL, respectively), which also suggested adrenal insufficiency. She was treated with hormone replacement therapy and antibiotics, successfully recovered from septic shock, and was withdrawn from dialysis. In empty sella syndrome, the somatotropic and gonadotropic axis are the most affected, followed by the thyrotropic and corticotropic axis. She did not present these abnormalities, which may suggest that empty sella syndrome is a separate pathology, and the axis suppression had occurred due to long-term steroid treatment. Diarrhea due to cytomegalovirus colitis might have induced steroid malabsorption and manifested adrenal insufficiency. Secondary adrenal insufficiency should be investigated as a cause of hyponatremia. It should always be borne in mind that diarrhea during oral steroid treatment may cause adrenal insufficiency associated with steroid malabsorption.

## Introduction

Renal transplant recipients are immunocompromised and have various complicating infections. However, atypical clinical presentations due to immunosuppression may make the diagnosis more difficult [1]. Renal transplant recipients are predisposed to develop hyponatremia because they are exposed to immunological, infectious, pharmacological, and oncologic disorders [2]. Empty sella syndrome is a neuroendocrine disorder in which the pituitary gland becomes flattened or shrunk. It is usually asymptomatic and can be an incidental radiological finding [3]. However, it can present with hypopituitarism, including secondary adrenal insufficiency, and chronic headache [4,5]. To our knowledge, there are few reports of renal transplant recipients with empty sella syndrome [6,7].

Here, we report the case of a post-transplant woman who presented with hyponatremia due to adrenal insufficiency caused by empty sella syndrome and steroid malabsorption. She also presented with various infections, such as cytomegalovirus (CMV) colitis, BK virus infections, and uroseptic shock.

## Case presentation

**Figure 1:**
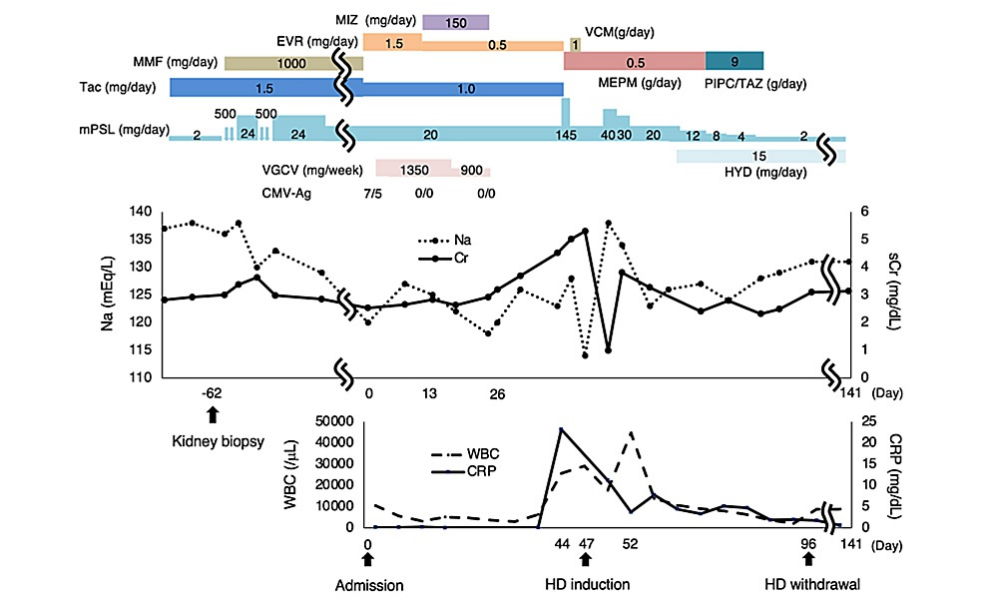
The clinical course of the patient. MIZ: mizoribine; EVR: everolimus; MMF: mycophenolate mofetil; Tac: tacrolimus; mPSL: methylprednisolone; VCM: vancomycin; MEPM: meropenem; PIPC/TAZ: piperacillin/tazobactam; CMV: cytomegalovirus; VGCV: valganciclovir; HYD: hydrocortisone

A 61-year-old woman who had undergone living-related kidney transplantation at the age of 57 years was admitted with diarrhea, anorexia, and headache for about a week. She had a history of hypertension, stroke, BK virus-associated nephropathy, CMV colitis, and chronic headache. There was no history suggesting Sheehan’s syndrome. She had been treated with 2 mg/day of methylprednisolone (mPSL) and 1.5 mg/day of tacrolimus (Tac). Sixty-two days before admission, a renal biopsy was performed because of an increase in urinary protein and serum creatinine levels. The kidney size was 99 mm × 44 mm. The patient was diagnosed with combined chronic active T-cell-mediated rejection (grade 1B) and chronic active antibody-mediated rejection. The clinical course of the patient is shown in Figure 1.

She was treated with intravenous mPSL pulse therapy (500 mg/day; two days, two times), followed by mPSL (24 mg/day), Tac (1.5 mg/day), and mycophenolate mofetil (MMF) (1,000 mg/day). Then, the dose of mPSL was reduced to 20 mg/day. On admission, her vital signs were as follows: blood pressure, 106/70 mmHg; temperature, 36.3°C; pulse, 77/minute; and respiratory rate, 18/minute. She presented with pale palpebral conjunctiva and extensive peripheral edema. The laboratory findings are summarized in Table 1.

**Table 1:** Laboratory data on admission. WBC: white blood cell; RBC: red blood cell; BUN: blood urea nitrogen; AST: aspartate aminotransferase; ALT: alanine aminotransferase; LDH: lactate dehydrogenase; γGTP: γ-glutamyl transpeptidase; ACTH: adrenocorticotropic hormone

Test name	Value
WBC (/μL)	10,500
RBC (/μL)	2.32 × 10^6^
Hemoglobin (g/dL)	7.1
Platelet count (/μL)	264 × 10^3^
Total protein (g/dL)	5.2
Albumin (g/dL)	3.3
BUN (mg/dL)	33.5
Creatinine (mg/dL)	2.53
Uric acid (mg/dL)	4.1
AST (IU/L)	10
ALT (IU/L)	8
LDH (IU/L)	225
γGTP (IU/L)	51
Sodium (mEq/L)	120
Potassium (mEq/L)	4.4
Chloride (mEq/L)	92
Calcium (mg/dL)	8.6
C-reactive protein (mg/dL)	0.12
ACTH (pg/mL)	2.6
Cortisol (μg/dL)	1.9
Serum osmolality (mOsm/kg)	271
Urine osmolality (mOsm/kg)	132
Urine sodium (mEq/L)	30

**Figure 2:**
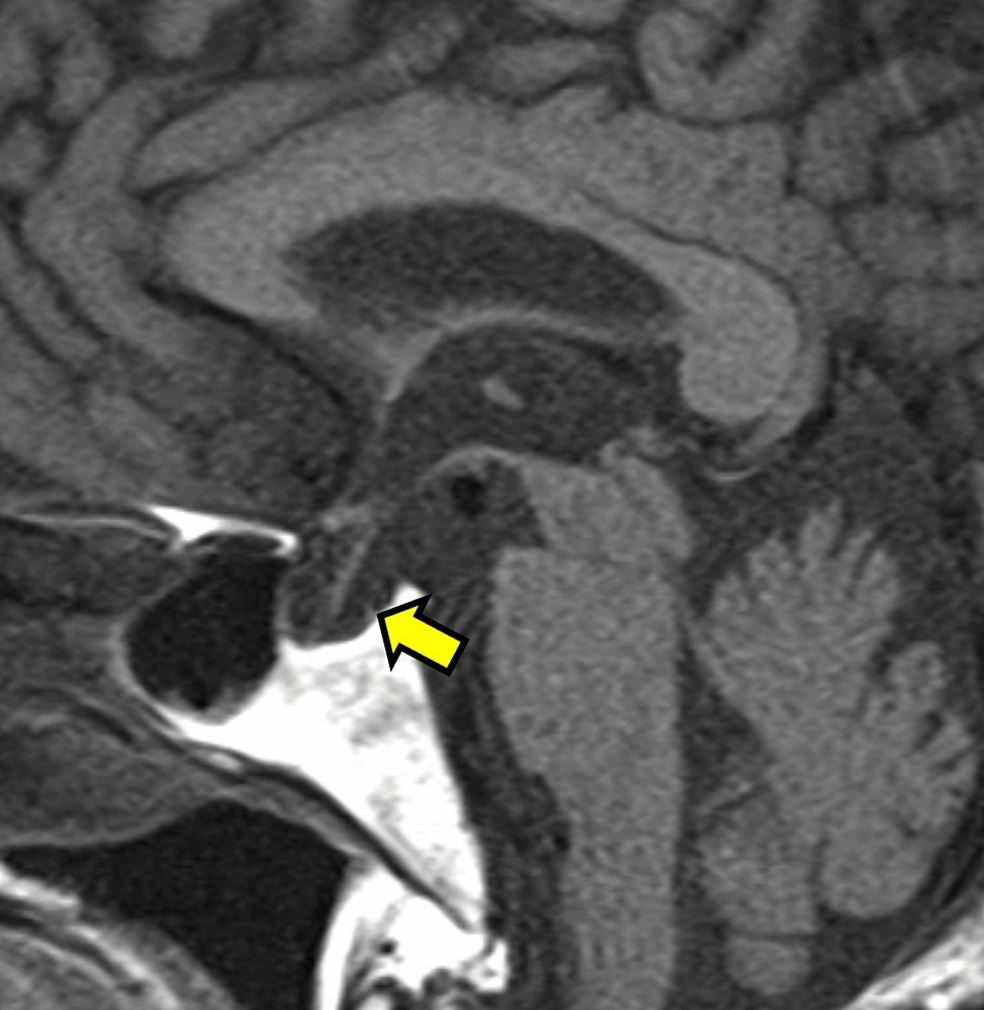
Brain magnetic resonance imaging revealing a thin and flat pituitary gland.

Laboratory findings revealed anemia and hyponatremia (7.1 g/dL and 120 mEq/L, respectively), with serum osmolality 271 of mOsm/kg, urine osmolality of 132 mOsm/kg, and urine sodium of 30 mEq/L. Her blood urea nitrogen and serum creatinine (sCr) levels were elevated (33.5 mg/dL and 2.53 mg/dL, respectively). The Tac trough level was 10.9 ng/mL. Endocrinological investigation revealed a low plasma cortisol level of .9 μg/dL (reference: 4.5-21.1 μg/dL), and a low adrenocorticotropic hormone (ACTH) level, of 2.6 pg/mL (reference: 7.2-63.3 pg/mL). Brain magnetic resonance imaging (MRI) to assess the hypothalamic-pituitary-adrenal axis revealed a thin and flat pituitary gland compatible with an empty sella (Figure 2).

Because diarrhea tended to improve upon admission, we continued mPSL at the same dose (20 mg/day). While we investigated the cause of diarrhea, CMV antigenemia was positive. A colonoscopy revealed edematous and erosive alterations in the mucosa between the cecum and the descending colon. She was diagnosed with CMV colitis recurrence and treated with valganciclovir (VGCV). On day 13, she tested negative for CMV antigenemia. We reduced the Tac dose, discontinued MMF owing to anemia, and started everolimus, followed by mizoribine (MIZ). In addition, because her SCr level gradually increased and the BK virus was detectable in both her blood and urine, we discontinued MIZ. Her serum sodium levels gradually declined to 118 mEq/L, and she experienced nausea and a headache on day 26 and was treated with intravenous 3% NaCl during the acute phase. On day 44, her consciousness was suddenly reduced, and her blood pressure could not be measured. Her blood tests revealed the following: white blood cell count (WBC), 25,700/μL; hemoglobin, 7.7 g/dL; platelet count, 43,000/μL; C-reactive protein (CRP), 23.2 mg/dL; sodium, 128 mEq/L; and sCr, 5.02 mg/dL (Table 2). Her serum procalcitonin and endotoxin levels were elevated (27.6 ng/mL and 2.8 pg/mL, respectively), and her fibrinogen, thrombin-antithrombin III complex, fibrin degradation products, and D-dimer levels were also markedly elevated. Endocrinological investigations revealed a plasma cortisol level of 5.2 μg/dL and an ACTH level of 13.5 pg/mL.

**Table 2:** Laboratory data. WBC: white blood cell; RBC: red blood cell; BUN: blood urea nitrogen; AST: aspartate aminotransferase; ALT: alanine aminotransferase; LDH: lactate dehydrogenase; γGTP: γ-glutamyl transpeptidase; TAT: thrombin-antithrombin III complex; FDP: fibrin degradation products; ACTH: adrenocorticotropic hormone

Test name	Value
WBC (/μL)	25,700
RBC (/μL)	2.31 × 10^6^
Hemoglobin (g/dL)	7.7
Platelet count (/μL)	43 × 10^3^
Total protein (g/dL)	3.7
Albumin (g/dL)	2.1
BUN (mg/dL)	61.9
Creatinine (mg/dL)	5.02
Uric acid (mg/dL)	8.5
AST (IU/L)	16
ALT (IU/L)	11
LDH (IU/L)	390
γGTP (IU/L)	90
Sodium (mEq/L)	128
Potassium (mEq/L)	3.4
Chloride (mEq/L)	99
Calcium (mg/dL)	8
C-reactive protein (mg/dL)	23.2
Procalcitonin (ng/mL)	27.6
Endotoxin (pg/mL)	2.8
Fibrinogen (mg/dL)	523
TAT (ng/mL)	20.1
FDP (μg/mL)	19
D-dimer (μg/mL)	8
ACTH (pg/mL)	13.5
Cortisol (μg/dL)	5.2

**Figure 3:**
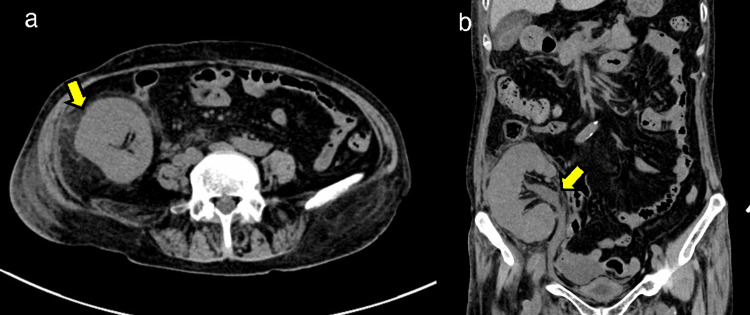
Abdominal computed tomography revealing swelling of the transplanted kidney, dilation of the ureter and renal pelvis, and perirenal fat stranding.

**Figure 4:**
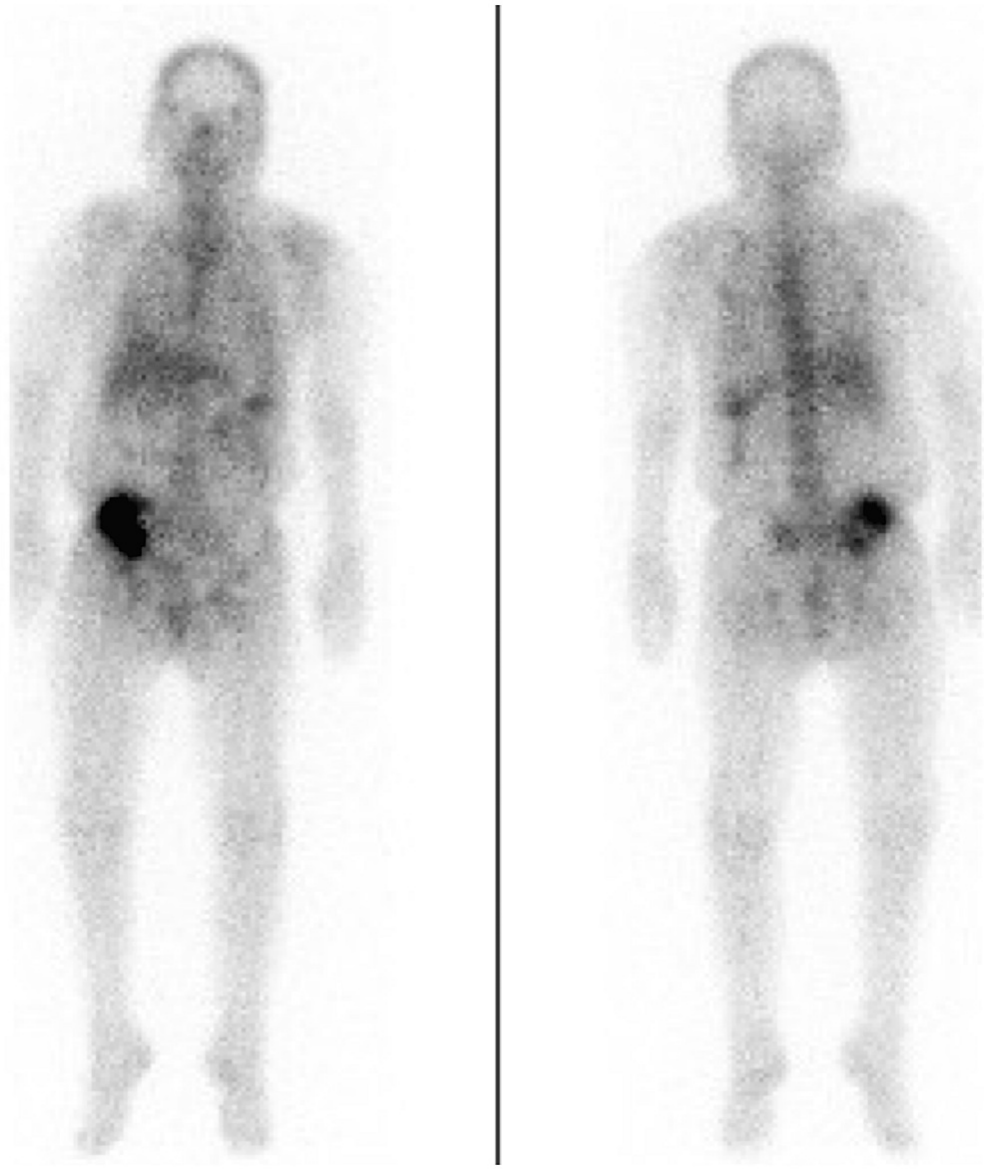
Gallium-67 scintigraphy revealing uptake in the transplanted kidney.

Her levels of thyroid-stimulating hormone, free T4, plasma renin activity, plasma aldosterone, luteinizing hormone, follicle-stimulating hormone, growth hormone, prolactin, and antidiuretic hormone were normal. Urinalysis revealed proteinuria, hematuria, pyuria, and bacteriuria. Abdominal computed tomography revealed swelling of the transplanted kidney (120 mm × 51 mm), dilation of the ureter and renal pelvis, and perirenal fat stranding (Figures 3a, 3b). Because there was a concern for other potential sites of infection, we performed gallium-67 scintigraphy, which revealed uptake in the transplanted kidney (Figure 4). Blood culture revealed *Klebsiella pneumoniae*.

Based on these results, the patient was diagnosed with septic shock and disseminated intravascular coagulation due to transplant pyelonephritis and was treated with meropenem. Intravenous mPSL was administered to treat adrenal insufficiency. However, the kidney function worsened. On day 47, hemodialysis was initiated owing to diuretic resistance and oliguria. Although her general condition tended to improve, her WBC again increased to 44,700/μL due to acute cholecystitis on day 52. She fasted and was administered intravenous fluids and antibiotics. She recovered and was withdrawn from dialysis on day 96 (Figure 1).

## Discussion

We report the case of a 61-year-old female renal transplant recipient presenting with hyponatremia due to secondary adrenal insufficiency. Empty sella syndrome was also diagnosed with low ACTH levels. Very few studies have reported how much the risk of adrenal insufficiency is increased in patients with empty sella syndrome. However, in empty sella syndrome, the somatotropic and gonadotropic axis are the most affected, followed by the thyrotropic and corticotropic axis [8]. These were spared in this patient, which may suggest that empty sella syndrome is a separate pathology, and the axis suppression had occurred due to long-term steroid treatment. All patients using corticosteroid therapy are at risk for adrenal insufficiency [9]. The clearance of prednisolone is reduced by 40% with uremia [10]. Therefore, in this case, it is likely that there was a relative adrenocortical hormone deficiency at the time of stress with sepsis.

She was treated with hormone replacement therapy and antibiotics, successfully recovered from septic shock, and was withdrawn from dialysis. Secondary adrenal insufficiency is easily overlooked among the many causes of hyponatremia. Low plasma cortisol and ACTH levels on admission suggest central hypoadrenalism, and the most common reason is exogenous glucocorticoid treatment [11]. We retrospectively investigated the sodium levels of this patient, who presented with recurrent hyponatremia. In this case, past recurrent hyponatremia and chronic headache might be related to empty sella syndrome, which manifested as adrenal insufficiency due to steroid malabsorption. It should always be borne in mind that diarrhea during oral steroid treatment may cause adrenal insufficiency associated with steroid malabsorption. It might be supported by the notion that enteric-coated prednisolone absorption was impaired in an asthmatic [12]. In such patients, changes in sodium levels should be closely monitored as a sign of adrenal insufficiency.

Post-transplant pyelonephritis is the most common bacterial infection in kidney transplant recipients [13] and is associated with an increased risk of graft loss and death [14]. CMV and BK virus infections are the major infection complications in renal transplant recipients [15]. Viral infections are biomarkers of excessive immunosuppression, and monitoring for reactivation of these viral infections should be routine. The maintenance TAC trough level should be within the range of 5 to 7 ng/mL after the first three months [16]. In this case, the TAC trough level was high when CMV colitis occurred, which may suggest the relationship between blood levels of immunosuppressants and CMV colitis. Patients with CMV reactivation should be actively screened for the BK virus [15]. In this case, we screened for and diagnosed the patient with BK virus infection and reduced immunosuppression.

Hyponatremia is associated with poorer outcomes in renal transplant recipients [17]. It is important to investigate the cause of hyponatremia and manage serum sodium levels together, especially in renal transplant recipients. We should also keep in mind that many classical symptoms of pyelonephritis are usually masked by immunosuppression [13]. Therefore, the first manifestation may be sepsis, as in the present case. Pyuria and bacteriuria should be investigated when renal transplant recipients have a fever or are suspected of having sepsis.

## Conclusions

Secondary adrenal insufficiency should be investigated as a cause of hyponatremia. It should always be borne in mind that diarrhea during oral steroid treatment may cause adrenal insufficiency associated with steroid malabsorption. Because renal transplant recipients with pyelonephritis are often clinically asymptomatic, we should investigate pyuria and bacteriuria when they have a fever.
